# Supplementary material for: A fully autonomous robotic ultrasound system for thyroid scanning
Source: Nat Commun. 2024 May 11;15:4004. doi: 10.1038/s41467-024-48421-y (PMC11519952; doi:10.1038/s41467-024-48421-y)
Supplement: Supplementary file 1 — Supplementary Information [file 41467_2024_48421_MOESM1_ESM.pdf]

A Fully Autonomous Robotic Ultrasound System for Thyroid Scanning

Kang Su[1†], Jingwei Liu[1†], Xiaoqi Ren[2,3†], Yingxiang Huo[2,3†], Guanglong Du[1*†], Wei Zhao[4], Xueqian Wang[5*], Bin Liang[6*], Di Li[7] and Peter Xiaoping Liu[8*]

[1]School of Computer Science and Engineering, South China University of Technology, Guangzhou, 510006, China.

[2]School of Future Technology, South China University of Technology, Guangzhou, 511442, China.

[3]Peng Cheng Laboratory, Shenzhen, 518000, China.

[4]Division of Vascular and Interventional Radiology, Nanfang Hospital Southern Medical University, Guangzhou, 510515, China.

[5]Tsinghua Shenzhen International Graduate School, Tsinghua University, Shenzhen, 518055, China.

[6]Department of Automation, Tsinghua University, Beijing, 100854, China.

[7]School of Mechanical and Automotive Engineering, South China University of Technology, Guangzhou, 510641, China.

[8]Department of Systems and Computer Engineering, Carleton University, Ottawa, K1S 5B6, Canada.

SUPPLEMENTARY INFORMATION

Supplementary Table 1

Table S1. In-plane scanning (IPS) phase evaluation from fourteen doctors' questionnaire regarding image quality, scanning completeness and centering performance.

Image quality	Scanning completeness					Centering performance				
(Mean±SD)	1	2	3	4	5	1	2	3	4	5
4.91±0.33	0	0	16	43	11	0	0	0	5	65
3.71±0.59	0	0	43	24	3	0	0	3	43	24
4.04±0.31	0	0	0	39	31	0	0	1	51	18
3.07±0.85	0	7	38	23	2	0	15	29	23	3
4.43±0.57	0	0	11	32	27	0	0	16	27	27
4.94±0.23	0	0	0	0	70	0	0	0	2	68
4.54±0.60	0	0	4	36	30	0	0	5	27	38
3.71±0.48	0	0	2	31	37	0	0	12	33	25
5.00±0.00	0	0	0	0	70	0	0	0	0	70
3.91±0.33	0	0	40	29	1	0	0	41	27	2
5.00±0.00	0	0	0	23	47	0	0	0	4	66
4.63±0.51	0	0	0	18	52	0	0	0	2	68
4.50±0.63	0	0	5	14	51	0	0	0	7	63
4.26±0.58	0	0	7	21	42	0	0	1	25	44

Supplementary Table 2

Table S2. Comparison of contact forces, probe motion transitions, total scanning time used for acquisition between the FARUS and professional doctors (Mean±SD).

	Robot	Doctor#1	Doctor#2	Doctor#3	Doctor#4	Doctor#5
F	2.5±0.5	2.0±0.9	2.5±0.7	2.4±0.6	2.5±0.7	2.5±0.7
V	3.0	11.7±2.1	11.4±1.8	12.6±2.5	12.2±2.2	12.4±1.9
ω	9.17	19.7±6.4	23.3±10.5	22.0±9.8	22.7±9.0	22.6±8.5
TST	213.0±85.3	69.9±26.6	73.8±27.1	58.2±26.6	65.2±32.3	64.1±21.5

Note: F: Force (N); V: Velocity (mm/s); ω: Angular velocity (degree/s); TST: Total scanning time (s).

Supplementary Table 3

Table S3. TRIPOD Checklist: Prediction Model Development and Validation. Items relevant only to the development of a prediction model are denoted by D, items relating solely to a validation of a prediction model are denoted by V, and items relating to both are denoted D;V.

Section/Topic	Item		Checklist Item	Page
**Title and abstract**				
Title	1	D;V	Identify the study as developing and/or validating a multivariable prediction model, the target population, and the outcome to be predicted.	Title page
Abstract	2	D;V	Provide a summary of objectives, study design, setting, participants, sample size, predictors, outcome, statistical analysis, results, and conclusions.	Abstract
**Introduction**				
Background and objectives	3a	D;V	Explain the medical context (including whether diagnostic or prognostic) and rationale for developing or validating the multivariable prediction model, including references to existing models.	Main
	3b	D;V	Specify the objectives, including whether the study describes the development or validation of the model or both.	Main
**Methods**				
Source of data	4a	D;V	Describe the study design or source of data (e.g., randomized trial, cohort, or registry data), separately for the development and	Methods: Human participants and safety

			validation data sets, if applicable.	Methods: Thyroid gland and nodule segmentation
	4b	D;V	Specify the key study dates, including start of accrual; end of accrual; and, if applicable, end of follow-up.	Methods: Human participants and safety
Participants	5a	D;V	Specify key elements of the study setting (e.g., primary care, secondary care, general population) including number and location of centres.	Methods: Human participants and safety
	5b	D;V	Describe eligibility criteria for participants.	Methods: Human participants and safety
	5c	D;V	Give details of treatments received, if relevant.	Not Applicable
Outcome	6a	D;V	Clearly define the outcome that is predicted by the prediction model, including how and when assessed.	Methods: Thyroid gland and nodule segmentation<br><br>Methods: Thyroid nodules scoring and classification
	6b	D;V	Report any actions to blind assessment of the outcome to be predicted.	Methods: Statistics and reproducibility
Predictors	7a	D;V	Clearly define all predictors used in developing or validating the multivariable prediction model, including how and when they were measured.	Methods: Thyroid gland and nodule segmentation
	7b	D;V	Report any actions to blind assessment of predictors	Methods: Statistics and reproducibility

			for the outcome and other predictors.	
Sample size	8	D;V	Explain how the study size was arrived at.	Methods: Statistics and reproducibility
Missing data	9	D;V	Describe how missing data were handled (e.g., complete-case analysis, single imputation, multiple imputation) with details of any imputation method.	Methods: Statistics and reproducibility
Statistical analysis methods	10a	D	Describe how predictors were handled in the analyses.	Methods: Thyroid gland and nodule segmentation<br><br>Methods: Thyroid nodules scoring and classification
	10b	D	Specify type of model, all model-building procedures (including any predictor selection), and method for internal validation.	Methods: Thyroid gland and nodule segmentation
	10c	V	For validation, describe how the predictions were calculated.	Methods: Thyroid gland and nodule segmentation<br><br>Methods: Thyroid nodules scoring and classification
	10d	D;V	Specify all measures used to assess model performance and, if relevant, to compare multiple models.	Results: Deep learning for gland and nodule segmentation<br><br>Results: ACR TI-RADS Risk

				Stratification using FARUS
	10e	V	Describe any model updating (e.g., recalibration) arising from the validation, if done.	Not Applicable
Risk groups	11	D;V	Provide details on how risk groups were created, if done.	Not Applicable
Development vs. validation	12	V	For validation, identify any differences from the development data in setting, eligibility criteria, outcome, and predictors.	Results: ACR TI-RADS Risk Stratification using FARUS
**Results**				
Participants	13a	D;V	Describe the flow of participants through the study, including the number of participants with and without the outcome and, if applicable, a summary of the follow-up time. A diagram may be helpful.	Methods: Human participants and safety
	13b	D;V	Describe the characteristics of the participants (basic demographics, clinical features, available predictors), including the number of participants with missing data for predictors and outcome.	Methods: Human participants and safety
	13c	V	For validation, show a comparison with the development data of the distribution of important variables (demographics, predictors and outcome).	Results: ACR TI-RADS Risk Stratification using FARUS
Model development	14a	D	Specify the number of participants and outcome events in each analysis.	Methods: Human participants and safety

	14b	D	If done, report the unadjusted association between each candidate predictor and outcome.	Not Applicable
Model specification	15a	D	Present the full prediction model to allow predictions for individuals (i.e., all regression coefficients, and model intercept or baseline survival at a given time point).	Not Applicable
	15b	D	Explain how to the use the prediction model.	Methods: Thyroid gland and nodule segmentation
Model performance	16	D;V	Report performance measures (with CIs) for the prediction model.	Results: Deep learning for gland and nodule segmentation
Model-updating	17	V	If done, report the results from any model updating (i.e., model specification, model performance).	Not Applicable
**Discussion**				
Limitations	18	D;V	Discuss any limitations of the study (such as nonrepresentative sample, few events per predictor, missing data).	Discussion
Interpretation	19a	V	For validation, discuss the results with reference to performance in the development data, and any other validation data.	Results: Deep learning for gland and nodule segmentation
	19b	D;V	Give an overall interpretation of the results, considering objectives, limitations, results from similar studies, and other relevant evidence.	Discussion

Implications	20	D;V	Discuss the potential clinical use of the model and implications for future research.	Discussion
**Other information**				
Supplementary information	21	D;V	Provide information about the availability of supplementary resources, such as study protocol, Web calculator, and data sets.	Supplementary Data 1
Funding	22	D;V	Give the source of funding and the role of the funders for the present study.	Acknowledgments

Supplementary Figure 1

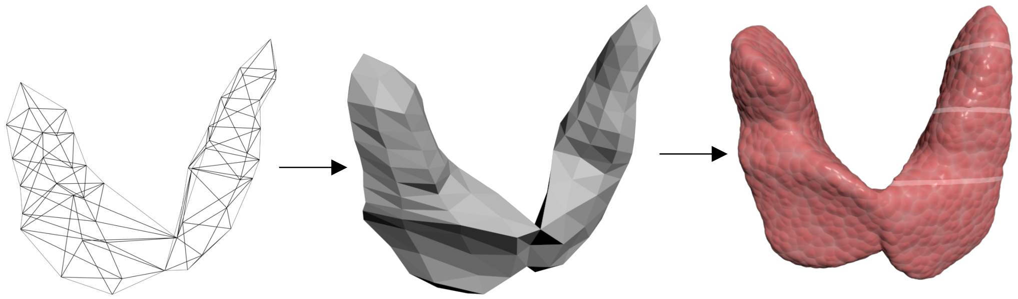

Supplementary Figure 1. Three-dimensional model of thyroid gland based on CT image reconstruction. From left to right are the mesh model, polygon model and rendering model.

Supplementary Figure 2

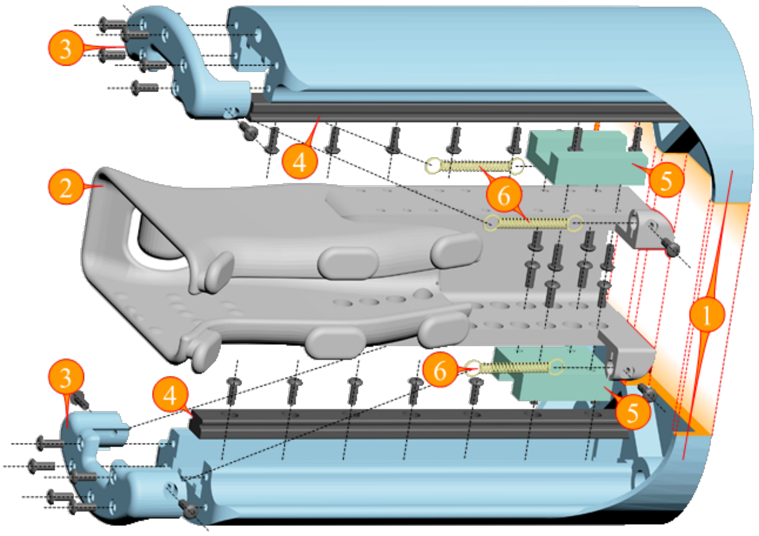

① Socket base ② US probe holder ③ Spring holder
④ Linear guide rail ⑤ Slider ⑥ Spring

Supplementary Figure 2. The 3D-printed resilient socket designed to accommodate the US probe comprises several components, including a socket base, probe holder, linear guide rail, slider, spring, and spring holder.
