# Supplementary material for: A fully autonomous robotic ultrasound system for thyroid scanning
Source: Nat Commun. 2024 May 11;15:4004. doi: 10.1038/s41467-024-48421-y (PMC11519952; doi:10.1038/s41467-024-48421-y)
Supplement: Supplementary file 3 — Description of Additional Supplementary Files [file 41467_2024_48421_MOESM3_ESM.docx]

**Description of Additional Supplementary Files**

**Supplementary Data 1: ﻿**Comparison of thyroid nodule scoring and recommended management between FARUS and doctor based on ACR TI-RADS

**Supplementary Movie 1:** The DQN learning model in FARUS demonstrates its ability to guide the robotic arm during thyroid scanning, even in the presence of patient movement or the absence of the thyroid gland.

**Supplementary Movie 2:** FARUS optimize the orientation of the ultrasound probe based on Bayesian optimization.

**Supplementary Movie 3:** The entire process of a robotic scanning of the thyroid gland with FARUS.
