# Supplementary material for: A fully autonomous robotic ultrasound system for thyroid scanning
Source: Nat Commun. 2024 May 11;15:4004. doi: 10.1038/s41467-024-48421-y (PMC11519952; doi:10.1038/s41467-024-48421-y)
Supplement: Supplementary file 4 — Supplementary Data 1 [file 41467_2024_48421_MOESM4_ESM.docx]

**Supplementary Data 1**

Data 1. Comparison of thyroid nodule scoring and recommended management between FARUS and doctor based on ACR TI-RADS. (Text in bold denotes scoring and recommended management from the doctor, while unbolded text denotes scoring and recommendations from the FARUS system. The columns labeled 'C,' 'E,' 'M,' 'S,' and 'E. F.' correspond to the criteria of 'Component,' 'Echogenicity,' 'Margin,' 'Shape,' and 'Echogenicity Foci,' respectively.)

| ID | Diagnosed by Doctor | Diagnosed by FARUS | C | E | M | S | E.F. | Recommendation |
| --- | --- | --- | --- | --- | --- | --- | --- | --- |
| #1 | 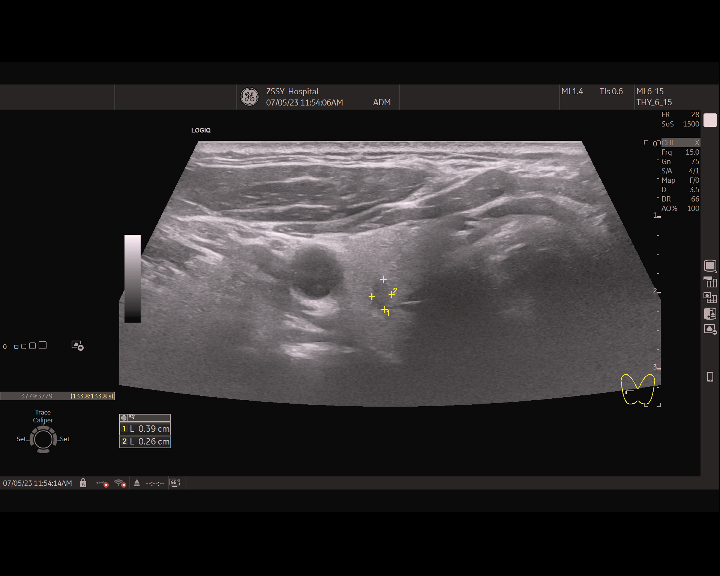 | 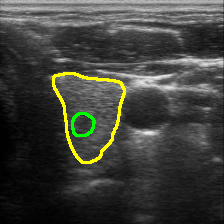 | 2 | 2 | 0 | 3 | 0 | No FNA |
|  |  |  | **2** | **2** | **0** | **3** | **0** | **No FNA** |
| #2 | 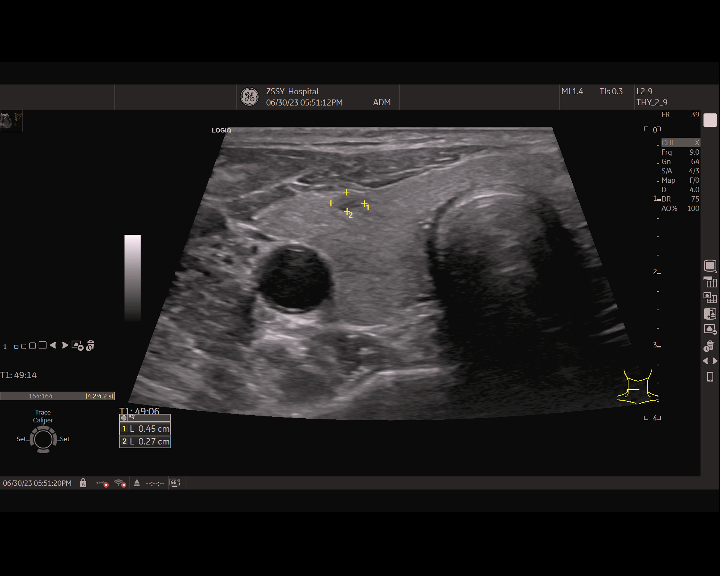 | 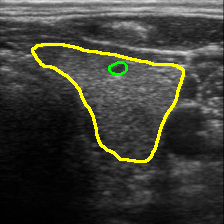 | 2 | 2 | 0 | 0 | 0 | No FNA |
|  |  |  | **1** | **1** | **0** | **0** | **0** | **No FNA** |
| #3 | 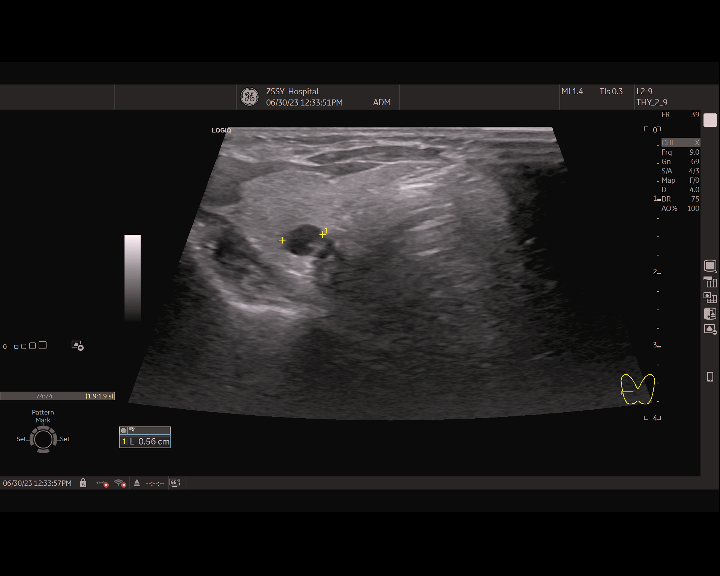 | 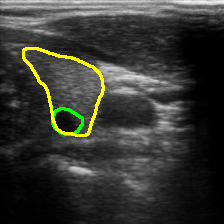 | 0 | 0 | 0 | 0 | 0 | No FNA |
|  |  |  | **0** | **0** | **0** | **0** | **0** | **No FNA** |
| #4 | 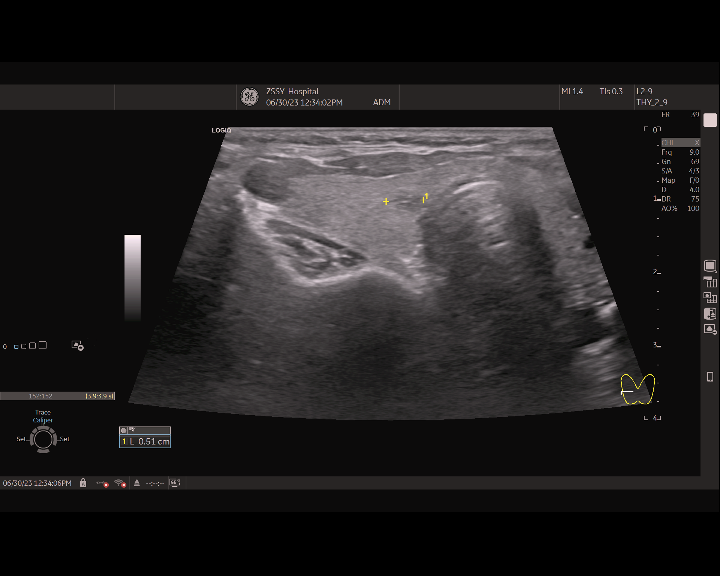 | 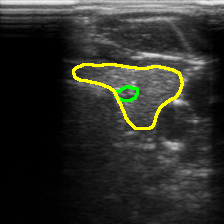 | 2 | 1 | 0 | 0 | 0 | No FNA |
|  |  |  | **1** | **1** | **0** | **0** | **0** | **No FNA** |
| #5 | 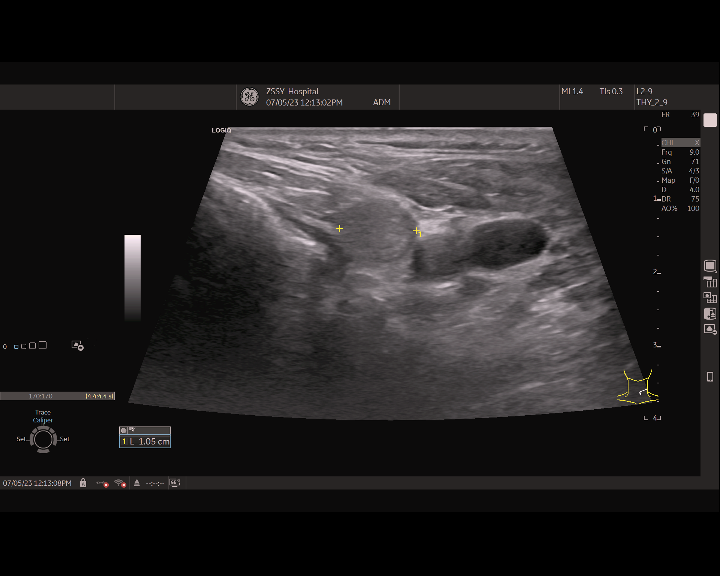 | 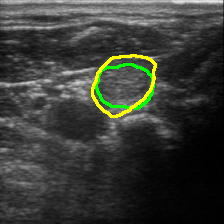 | 2 | 2 | 0 | 0 | 0 | Follow-up |
|  |  |  | **2** | **1** | **0** | **0** | **0** | **No FNA** |
| #6 | 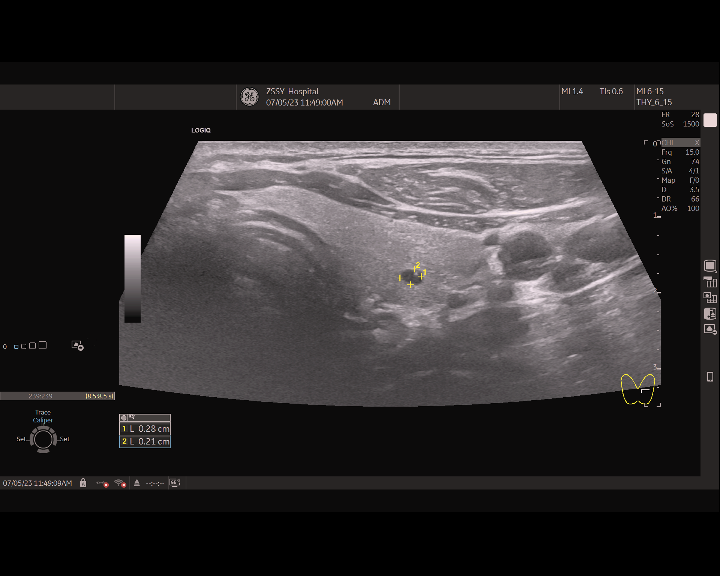 | 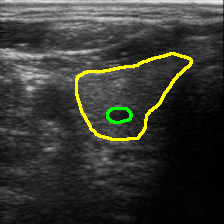 | 2 | 2 | 0 | 0 | 0 | No FNA |
|  |  |  | **0** | **0** | **0** | **0** | **0** | **No FNA** |
| #7 | 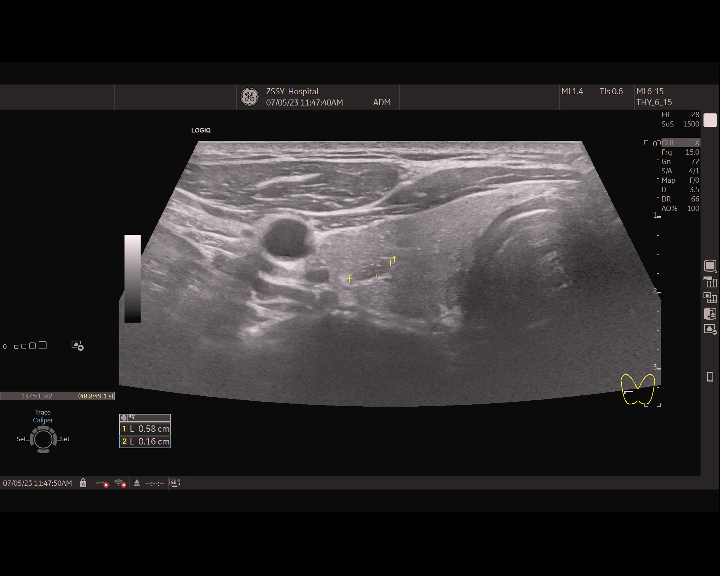 | 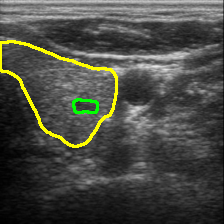 | 2 | 1 | 0 | 0 | 0 | No FNA |
|  |  |  | **1** | **2** | **0** | **0** | **0** | **No FNA** |
| #8 | 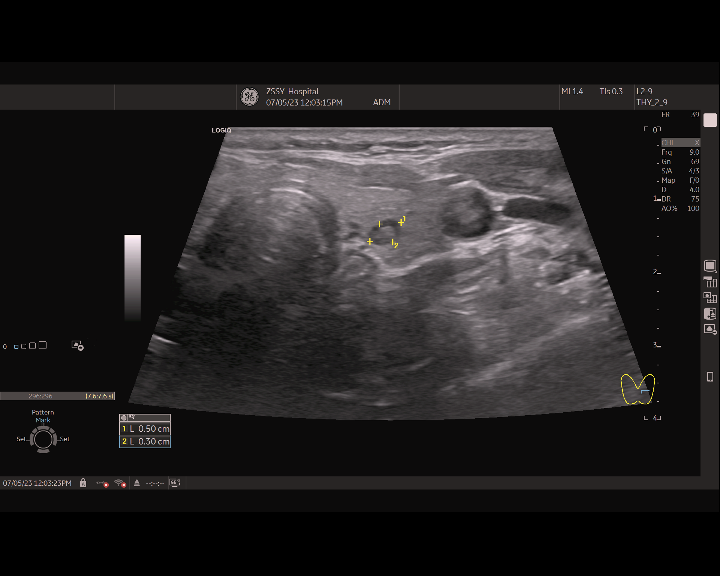 | 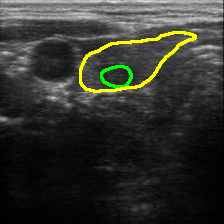 | 1 | 2 | 0 | 0 | 0 | No FNA |
|  |  |  | **1** | **1** | **0** | **0** | **0** | **No FNA** |
| #9 | 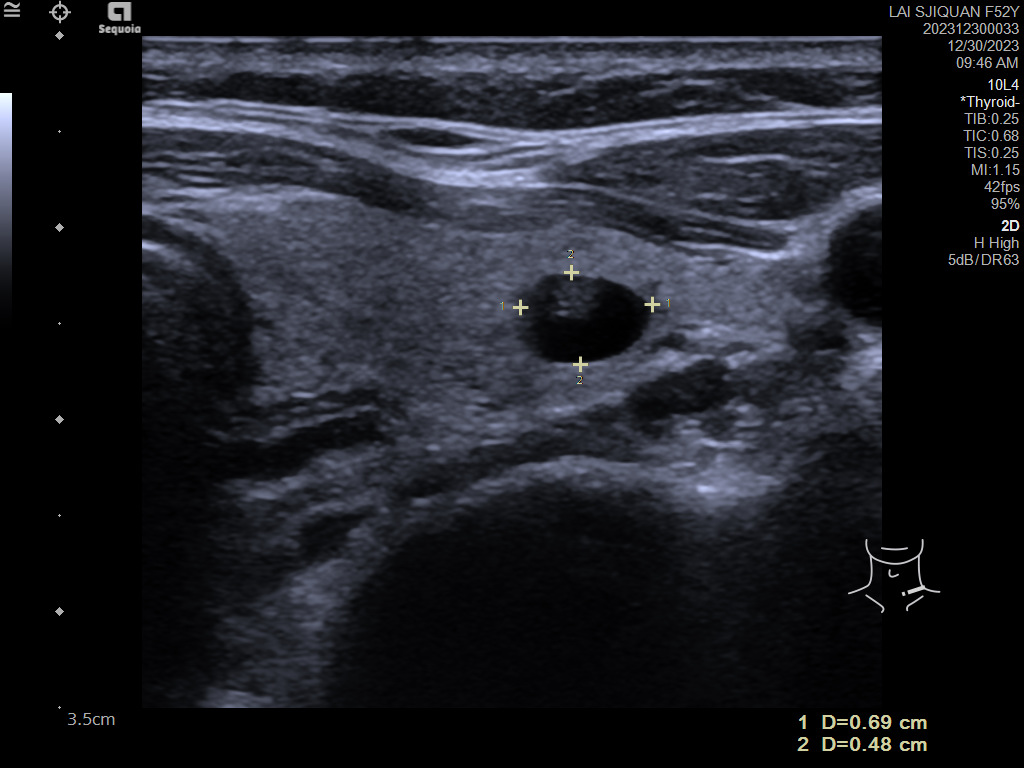 | 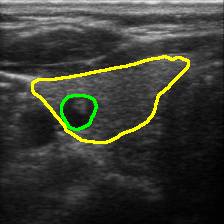 | 1 | 2 | 0 | 0 | 0 | No FNA |
|  |  |  | **1** | **2** | **0** | **0** | **0** | **No FNA** |
| #10 | 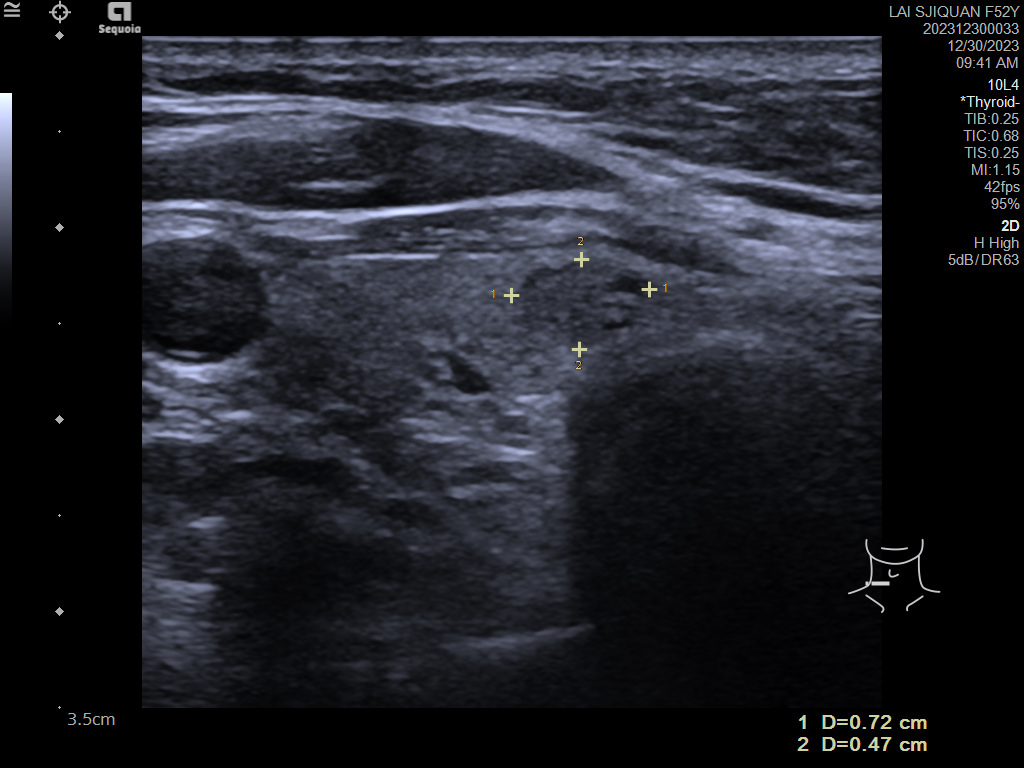 | 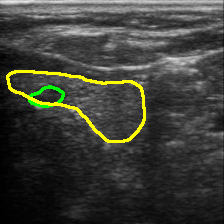 | 2 | 2 | 0 | 0 | 0 | No FNA |
|  |  |  | **1** | **2** | **0** | **0** | **0** | **No FNA** |
| #11 | 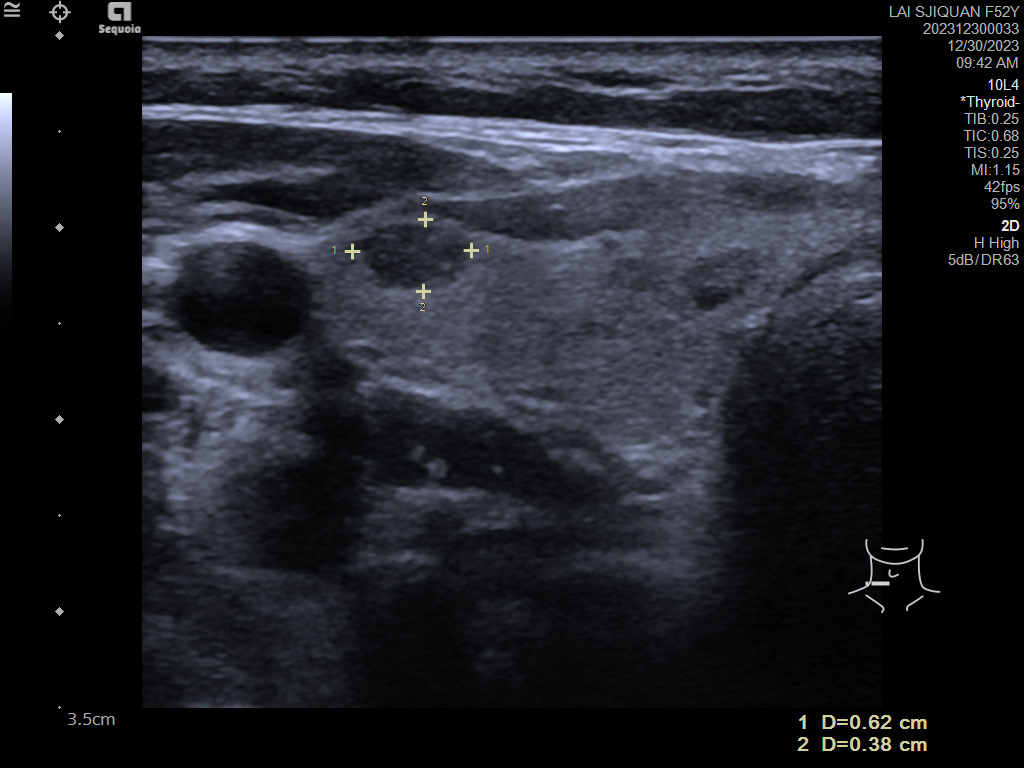 | 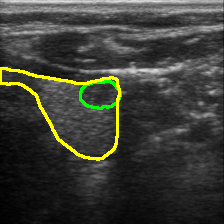 | 2 | 2 | 0 | 0 | 0 | No FNA |
|  |  |  | **1** | **1** | **0** | **0** | **0** | **No FNA** |
| #12 | 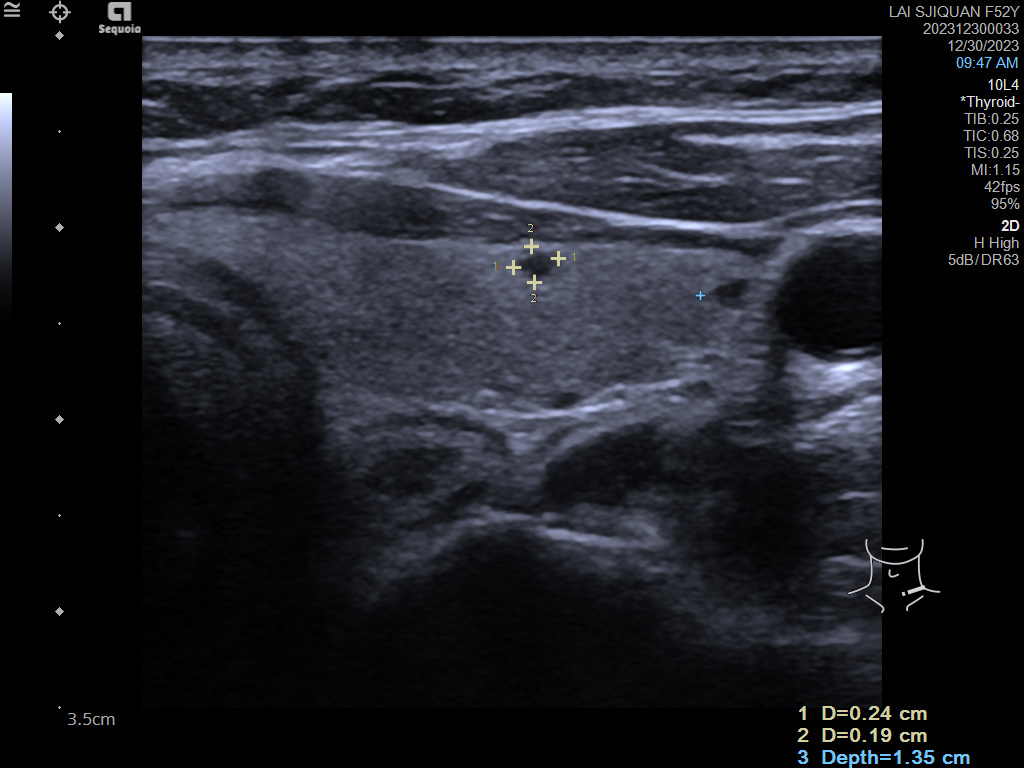 | 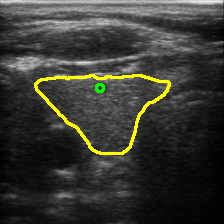 | 2 | 2 | 0 | 0 | 0 | No FNA |
|  |  |  | **1** | **1** | **0** | **0** | **0** | **No FNA** |
| #13 | 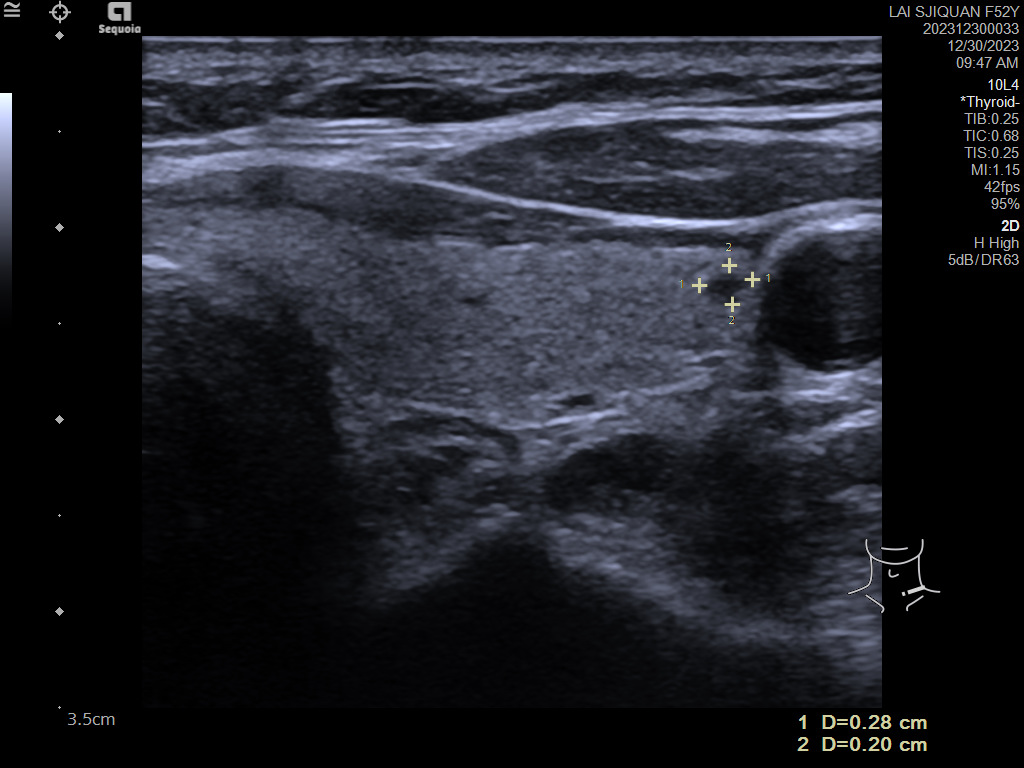 | 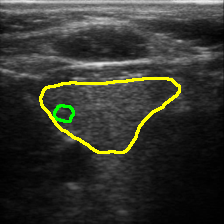 | 2 | 2 | 0 | 0 | 0 | No FNA |
|  |  |  | **1** | **1** | **0** | **0** | **0** | **No FNA** |
| #14 | 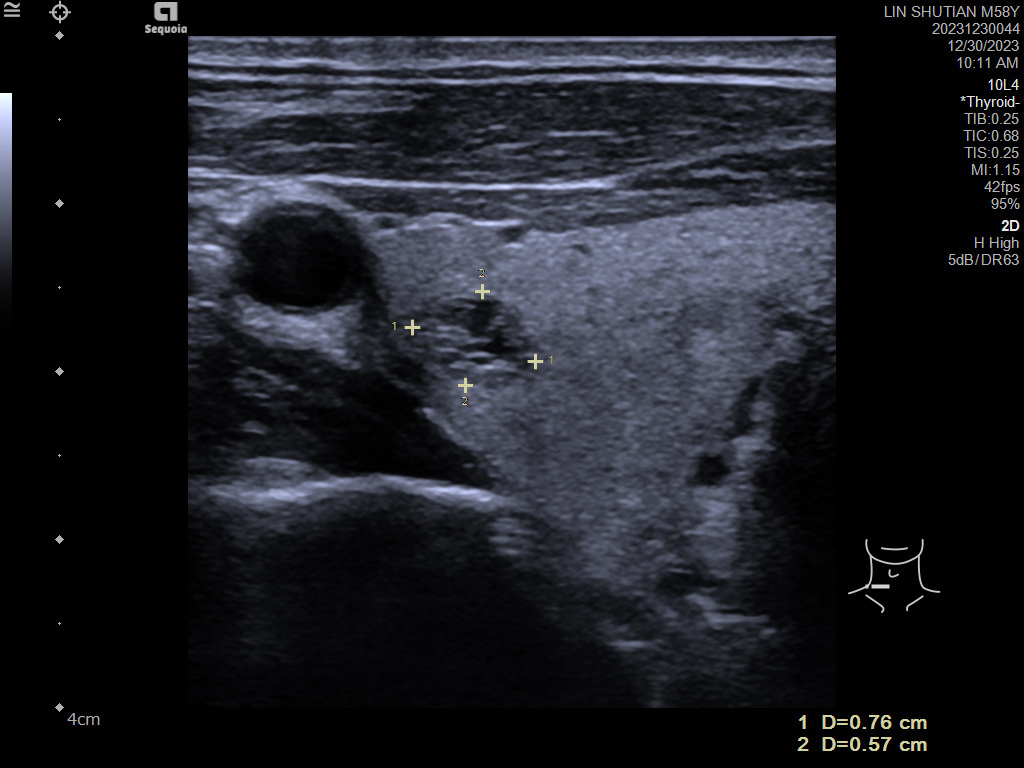 | 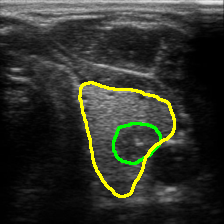 | 1 | 2 | 0 | 0 | 0 | No FNA |
|  |  |  | **1** | **1** | **0** | **0** | **0** | **No FNA** |
| #15 | 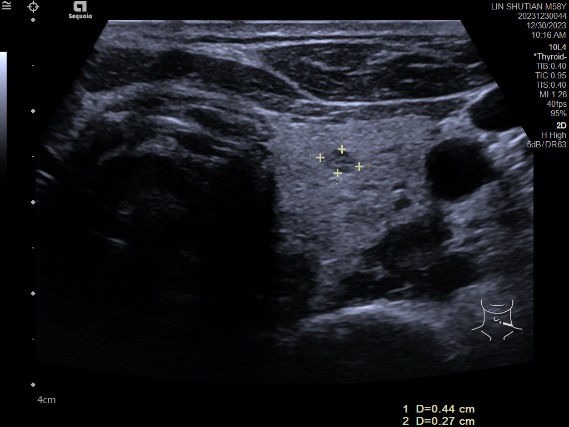 | 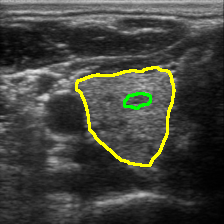 | 1 | 1 | 0 | 0 | 0 | No FNA |
|  |  |  | **1** | **1** | **0** | **0** | **0** | **No FNA** |
| #16 | 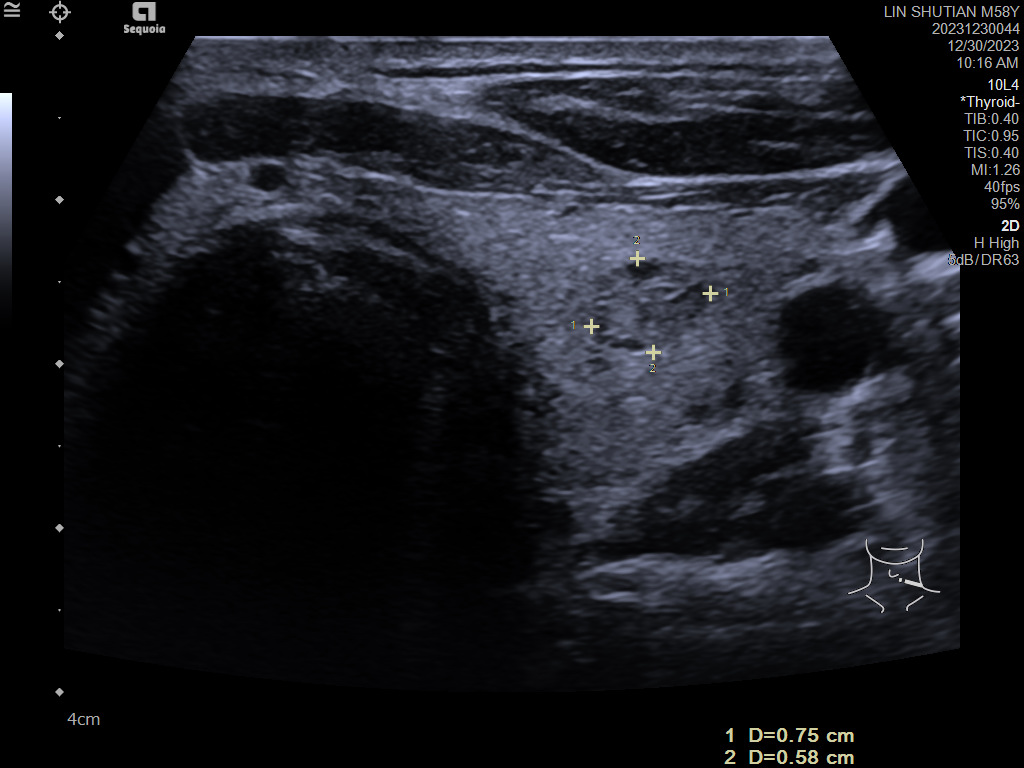 | 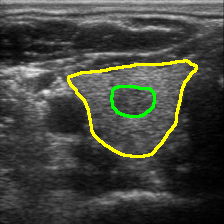 | 1 | 1 | 0 | 0 | 0 | No FNA |
|  |  |  | **1** | **1** | **0** | **0** | **0** | **No FNA** |
| #17  #18 | 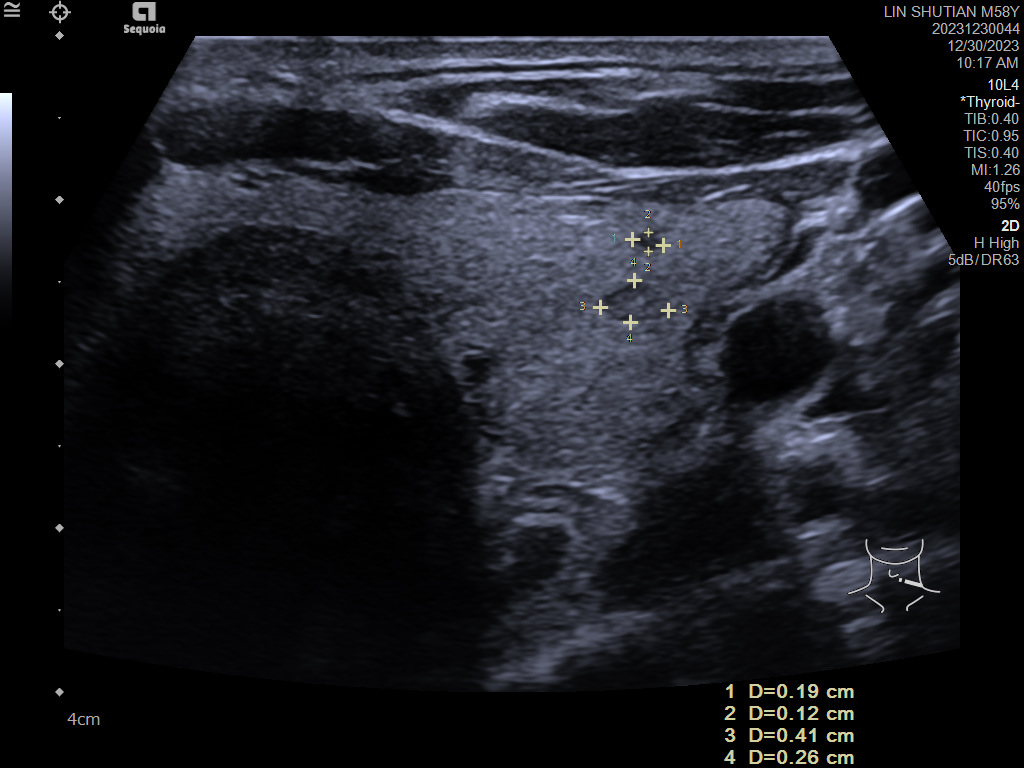 | 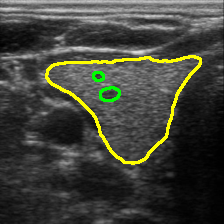 | 1 | 1 | 0 | 0 | 0 | No FNA |
|  |  |  | **1** | **1** | **0** | **0** | **0** | **No FNA** |
| #19 | 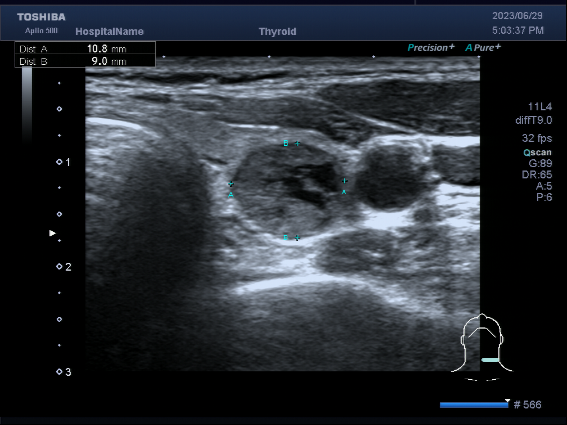 | 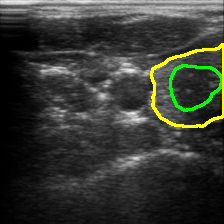 | 1 | 2 | 0 | 0 | 0 | Follow-up |
|  |  |  | **1** | **2** | **0** | **0** | **0** | **Follow-up** |
| #20 | 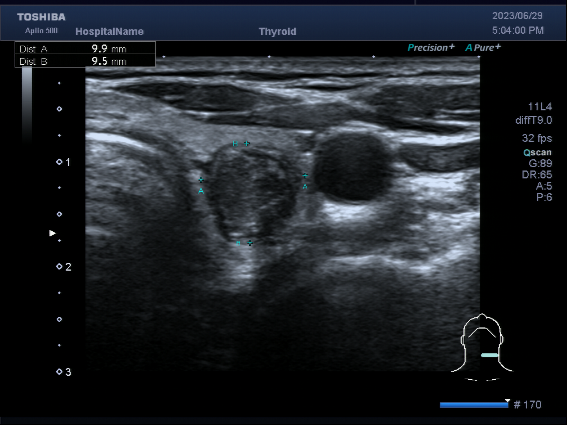 | 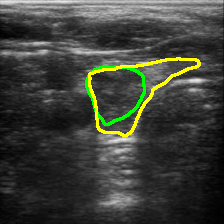 | 1 | 2 | 0 | 0 | 2 | FNA |
|  |  |  | **1** | **2** | **0** | **0** | **2** | **FNA** |
| #21 | 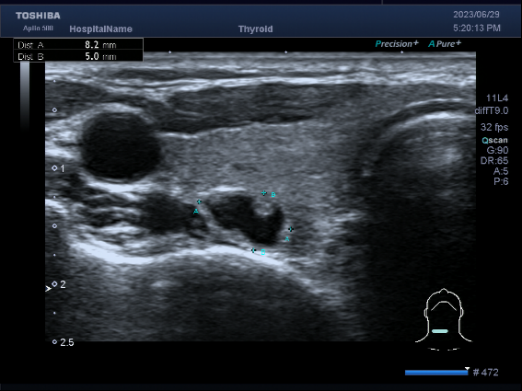 | 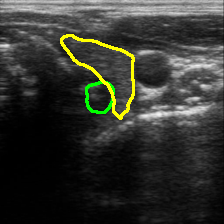 | 1 | 2 | 0 | 0 | 0 | No FNA |
|  |  |  | **1** | **1** | **0** | **0** | **0** | **No FNA** |
| #22 | 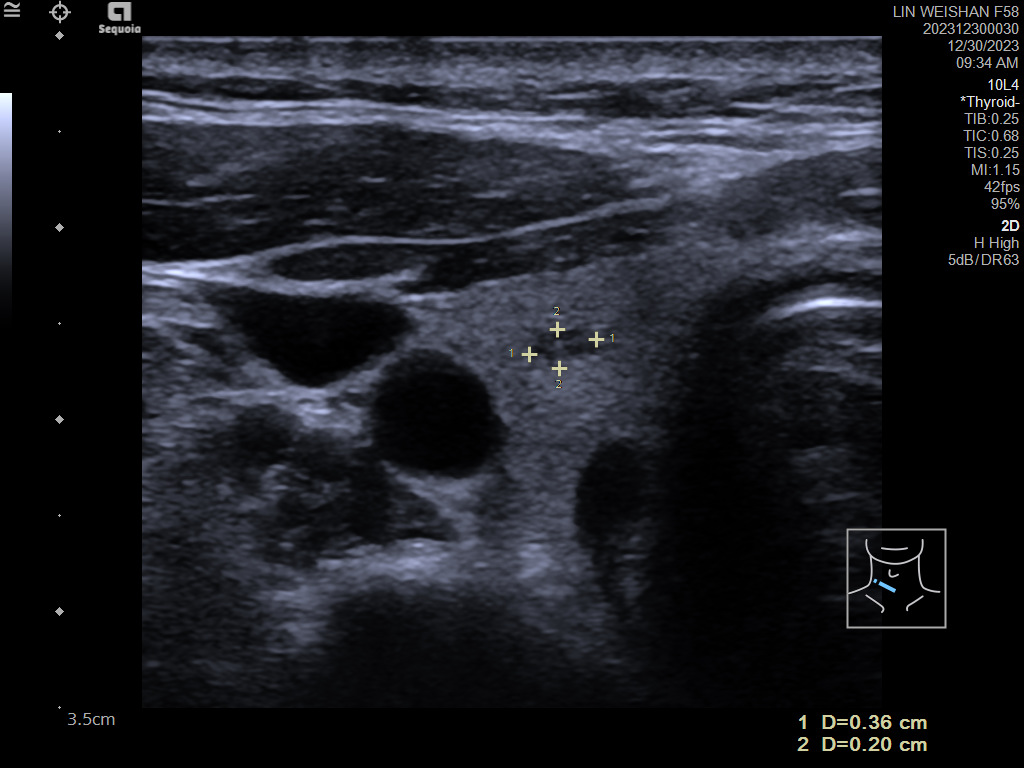 | 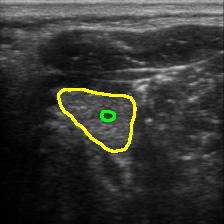 | 1 | 2 | 0 | 0 | 0 | No FNA |
|  |  |  | **1** | **1** | **0** | **0** | **0** | **No FNA** |
| #23 | 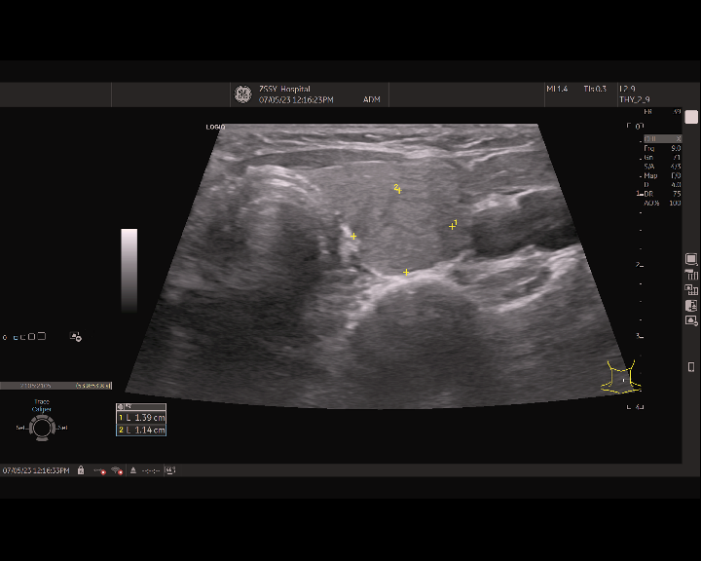 | 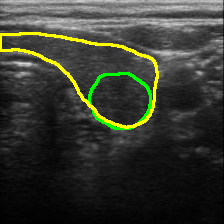 | 2 | 1 | 0 | 0 | 0 | No FNA |
|  |  |  | **2** | **1** | **0** | **0** | **0** | **No FNA** |
| #24 | 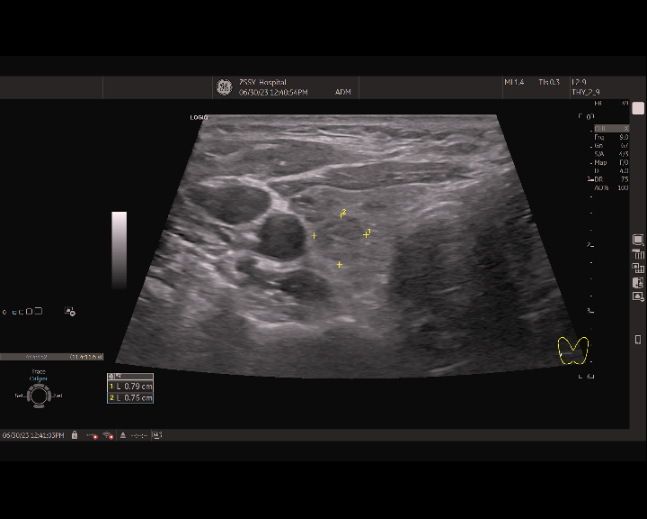 | 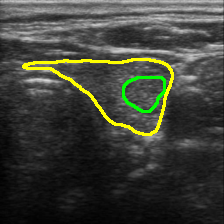 | 2 | 1 | 0 | 0 | 0 | No FNA |
|  |  |  | **1** | **1** | **0** | **0** | **0** | **No FNA** |
| #25 | 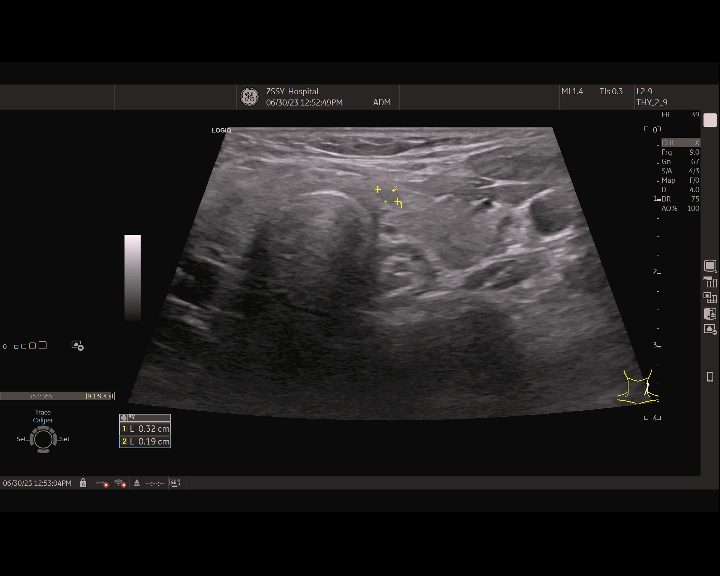 | Missed | **2** | **2** | **0** | **0** | **0** | **No FNA** |
| #26 | 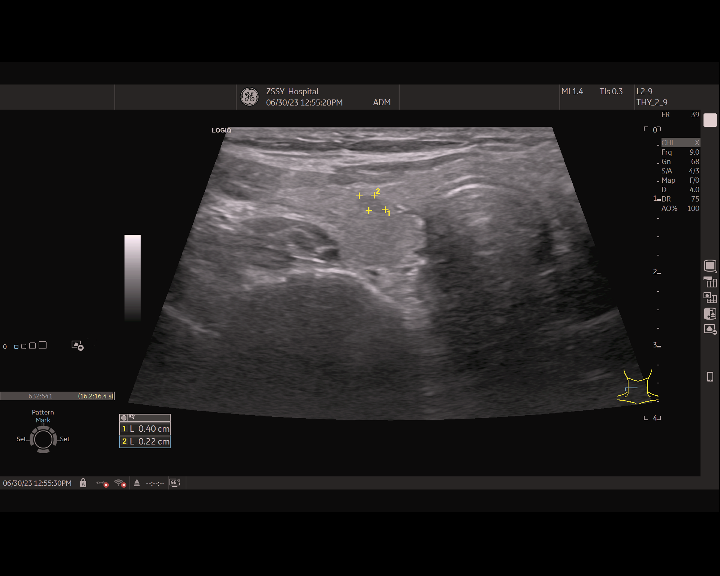 | Missed | **2** | **1** | **0** | **0** | **0** | **No FNA** |
| #27 | 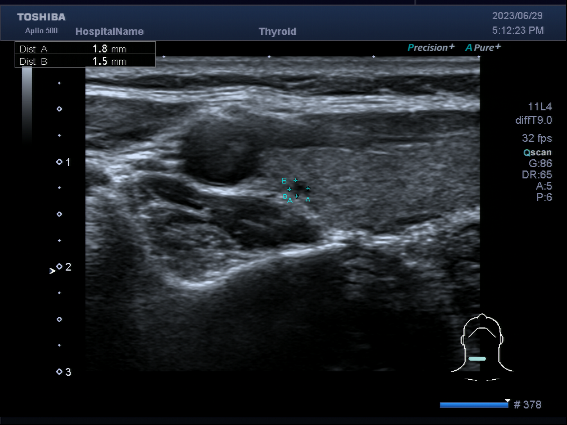 | Missed | **1** | **2** | **0** | **0** | **0** | **No FNA** |
| #28 | 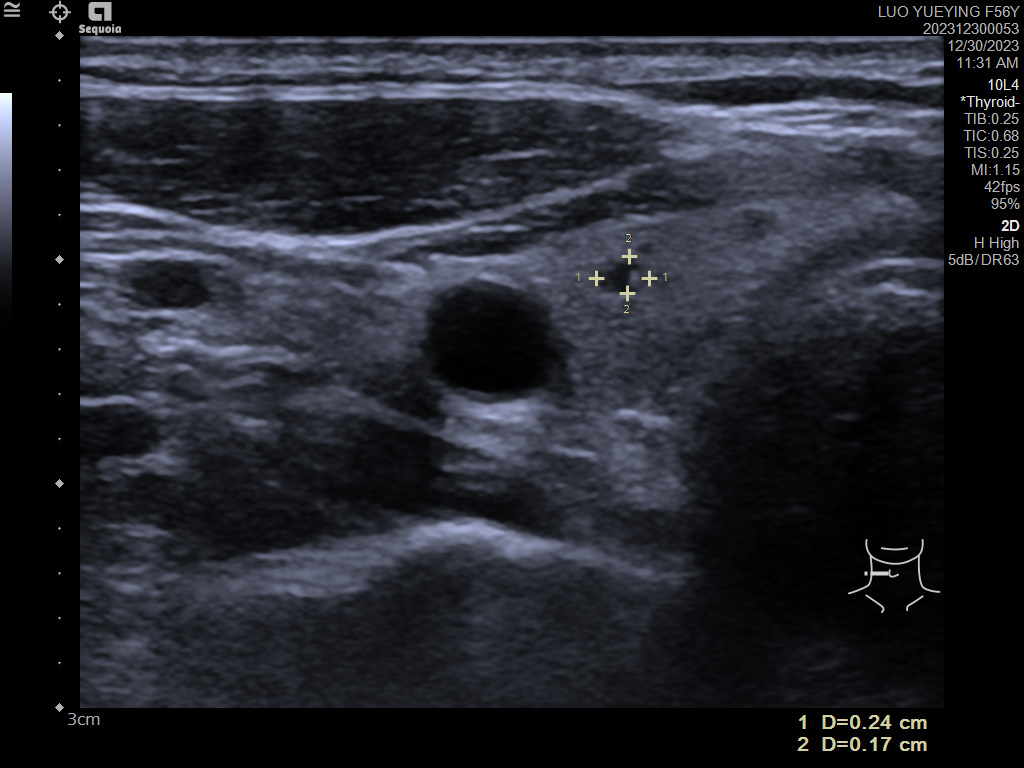 | Missed | **1** | **1** | **0** | **0** | **0** | **No FNA** |
| #29 | 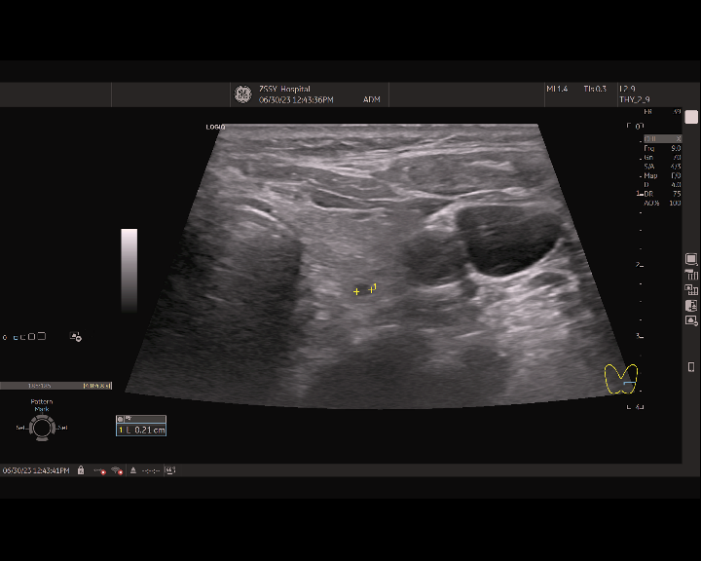 | Missed | **0** | **0** | **0** | **0** | **0** | **No FNA** |
| #30 | 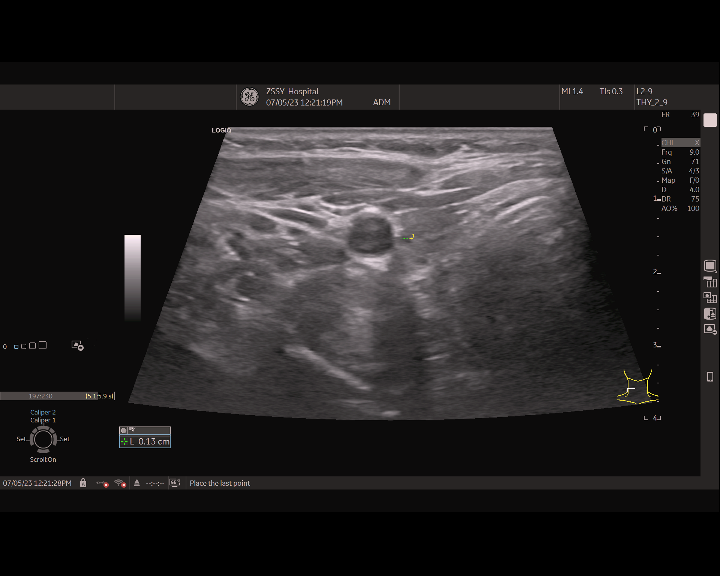 | Missed | **0** | **0** | **0** | **0** | **0** | **No FNA** |
| #31 | 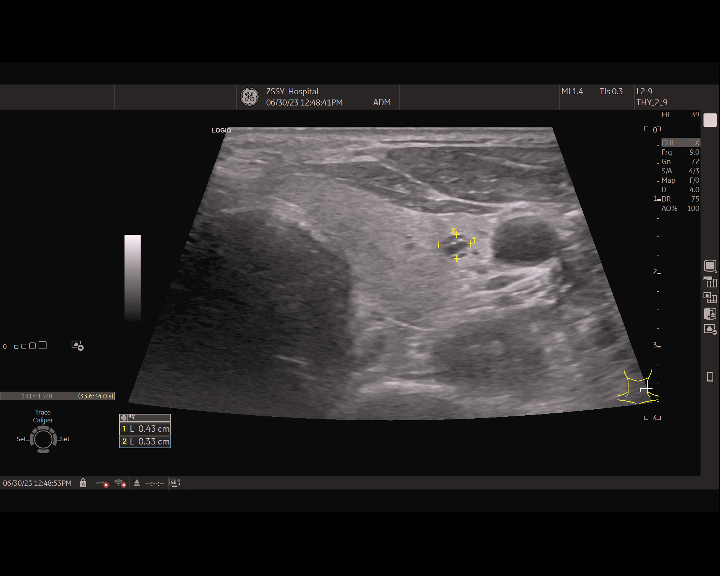 | Missed | **2** | **1** | **0** | **0** | **0** | **No FNA** |
| #32 | 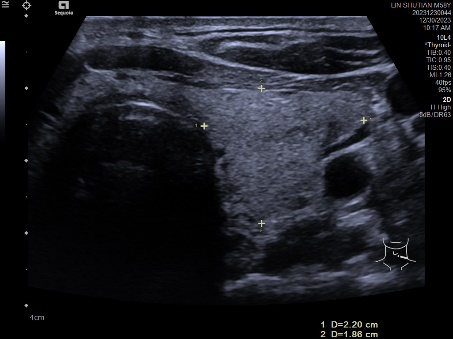 | Missed | **2** | **1** | **0** | **0** | **0** | **No FNA** |
| #33 | Not found | 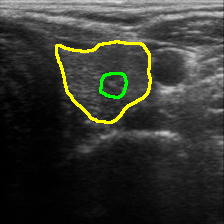 | 2 | 1 | 0 | 0 | 0 | No FNA |
| #34 | Not found | 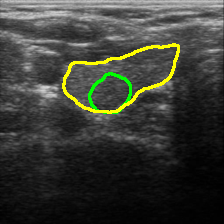 | 2 | 2 | 0 | 0 | 0 | No FNA |
| #35 | Not found | 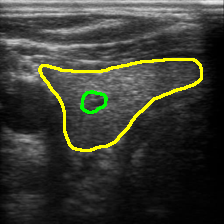 | 2 | 1 | 0 | 0 | 0 | No FNA |
| #36 | Not found | 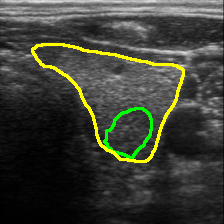 | 1 | 2 | 0 | 3 | 0 | Follow-up |
