# Supplementary material for: A fully autonomous robotic ultrasound system for thyroid scanning
Source: Nat Commun. 2024 May 11;15:4004. doi: 10.1038/s41467-024-48421-y (PMC11519952; doi:10.1038/s41467-024-48421-y)
Supplement: Supplementary file 8 — Reporting Summary [file 41467_2024_48421_MOESM8_ESM.pdf]

nature portfolio

Corresponding author(s): Guanglong Du, Xueqian Wang, Bin Liang, Peter Xiaoping Liu and NCOMMS-23-00951B

Last updated by author(s): Feb 14, 2024

Reporting Summary

Nature Portfolio wishes to improve the reproducibility of the work that we publish. This form provides structure for consistency and transparency in reporting. For further information on Nature Portfolio policies, see our Editorial Policies and the Editorial Policy Checklist.

Statistics

For all statistical analyses, confirm that the following items are present in the figure legend, table legend, main text, or Methods section.

n/a	Confirmed	
☐	☒	The exact sample size (*n*) for each experimental group/condition, given as a discrete number and unit of measurement
☐	☒	A statement on whether measurements were taken from distinct samples or whether the same sample was measured repeatedly
☐	☒	The statistical test(s) used AND whether they are one- or two-sided<br>*Only common tests should be described solely by name; describe more complex techniques in the Methods section.*
☒	☐	A description of all covariates tested
☒	☐	A description of any assumptions or corrections, such as tests of normality and adjustment for multiple comparisons
☐	☒	A full description of the statistical parameters including central tendency (e.g. means) or other basic estimates (e.g. regression coefficient) AND variation (e.g. standard deviation) or associated estimates of uncertainty (e.g. confidence intervals)
☒	☐	For null hypothesis testing, the test statistic (e.g. *F*, *t*, *r*) with confidence intervals, effect sizes, degrees of freedom and *P* value noted<br>*Give P values as exact values whenever suitable.*
☐	☒	For Bayesian analysis, information on the choice of priors and Markov chain Monte Carlo settings
☒	☐	For hierarchical and complex designs, identification of the appropriate level for tests and full reporting of outcomes
☐	☒	Estimates of effect sizes (e.g. Cohen's *d*, Pearson's *r*), indicating how they were calculated

Our web collection on statistics for biologists contains articles on many of the points above.

Software and code

Policy information about availability of computer code

Data collection	1. Kinect Azure SDK (vl.4.1): 2D view of scene acquisition<br>2. Azure Kinect Body Tracking SDK (V1.1.2): Human skeleton joints<br>3. ft300python (V1.0.2): Force/Torque data<br>4. Costume code: Robotic Scanning
Data analysis	Software used for data analysis:<br>1. Bayesian Optimization kit(V1.4.3) : Ultrasound probe orientation optimization;<br>2. Pytorch 1.10.0+CUDA 11.3: Al models training;<br>3. Statical Analysis: math3d 3.4.1, mmcv 2.0.0, numpy 1.23.5, opencv-python 4.6.0.66, pandas 1.5.2, scikit-learn 1.2.0, scikit-image 0.20.0<br>4. Costume code is available at: https://github.com/Ciel04sk/SCUT_Thyroid_DataSet/tree/main

For manuscripts utilizing custom algorithms or software that are central to the research but not yet described in published literature, software must be made available to editors and reviewers. We strongly encourage code deposition in a community repository (e.g. GitHub). See the Nature Portfolio guidelines for submitting code & software for further information.

Data

Policy information about availability of data

All manuscripts must include a data availability statement. This statement should provide the following information, where applicable:

- Accession codes, unique identifiers, or web links for publicly available datasets
- A description of any restrictions on data availability
- For clinical datasets or third party data, please ensure that the statement adheres to our policy

The main data generated in this study have been deposited in the github: https://github.com/Ciel04sk/SCUT_Thyroid_DataSet/tree/main. The graph and table data within this work are provided in the Supplementary Information/Source Data file. Source data are provided with this paper.

Research involving human participants, their data, or biological material

Policy information about studies with human participants or human data. See also policy information about sex, gender (identity/presentation), and sexual orientation and race, ethnicity and racism.

Reporting on sex and gender	To assess the robotic image quality of ultrasound scans, we employed the FARUS system to autonomously scan 70 college students (20–30 years of age, 19 females, 41 males). To investigate the diagnostic outcomes generated by our robotic system for thyroid nodules against the established hospital benchmark, ultrasound thyroid images were obtained from 19 patients (age 53.05 ±5.90 years old, 12 females, 7 males). Then the American College of Radiology Thyroid Imaging Reporting and Data System (ACR TI-RADS) scoring method was applied to classify nodules as either benign or malignant.
Reporting on race, ethnicity, or other socially relevant groupings	N/A.
Population characteristics	The participants were over 18 years old, and of both sexes. None of the participants had following cases: (1) neck trauma or failure to heal after surgery; (2) patients who cannot maintain a stable head position;(3) history of ultrasound glue allergies; (4) history of surgical resection of both thyroid lobes.
Recruitment	With the approval of the ethical review committee, we recruited of three distinct groups of participants. The participants were over 18 years old, and of both sexes. None of the participants had following cases: (1) neck trauma or failure to heal after surgery; (2) inability to maintain a stable head position;(3) history of US gel allergies; (4) history of surgical resection of both thyroid lobes. The first group predominantly comprised college students, and we manually collected thyroid US data from 66 volunteers within this group using handheld US equipment. Simultaneously, we employed FARUS system to autonomously scan 70 volunteers (20–30 years of age, 19 females, 41 males), 13 of whom were scanned manually by 5 doctors. To address the limitations of handheld US equipment in accurately diagnosing nodules, we opted to employ portable US equipment to gather two additional sets of data. The second set of data was obtained from thyroid US scans of 29 middle-aged and elderly individuals within the community, chosen specifically to facilitate the training of the nodule segmentation network. Finally, the third group was composed of 19 patients (age 53.05 ±5.90 years old, 12 females, 7 males) who underwent robotic thyroid scanning. This group played a pivotal role in verifying the diagnostic performance of our FARUS system. The recruitment bias primarily arises from the geographic scope of recruitment, limited to a specific region in China.
Ethics oversight	All experiments with human participants were performed with the approval of the Guangzhou First People's Hospital Review Board (K-2021-131-04). Our researchers explained the entire process to all participants, and all participants signed an informed consent form.

Note that full information on the approval of the study protocol must also be provided in the manuscript.

Field-specific reporting

Please select the one below that is the best fit for your research. If you are not sure, read the appropriate sections before making your selection.

☒ Life sciences ☐ Behavioural & social sciences ☐ Ecological, evolutionary & environmental sciences

For a reference copy of the document with all sections, see nature.com/documents/nr-reporting-summary-flat.pdf

Life sciences study design

All studies must disclose on these points even when the disclosure is negative.

Sample size	No statistical method was used to predetermine sample size. To assess the robotic image quality of ultrasound scans, we employed the FARUS system to autonomously scan 70 college students (n=70). Additionally, to investigate the diagnostic performance of our FARUS system, robotic thyroid scanning was performed on nineteen patients (n=19).
Data exclusions	No data were excluded from the analyses.
Replication	The robotic thyroid scans were successfully conducted on 89 participants, including 70 college students and 19 patients.
Randomization	NA.

Blinding	NA.

Reporting for specific materials, systems and methods

We require information from authors about some types of materials, experimental systems and methods used in many studies. Here, indicate whether each material, system or method listed is relevant to your study. If you are not sure if a list item applies to your research, read the appropriate section before selecting a response.

Materials & experimental systems

n/a	Involved in the study
☒	☐ Antibodies
☒	☐ Eukaryotic cell lines
☒	☐ Palaeontology and archaeology
☒	☐ Animals and other organisms
☒	☐ Clinical data
☒	☐ Dual use research of concern
☒	☐ Plants

Methods

n/a	Involved in the study
☒	☐ ChIP-seq
☒	☐ Flow cytometry
☒	☐ MRI-based neuroimaging

Plants

Seed stocks	NA.
Novel plant genotypes	NA.
Authentication	NA.
